# Major depressive symptoms in breast cancer patients with ovarian function suppression: a cross-sectional study comparing ovarian ablation and gonadotropin-releasing hormone agonists

**DOI:** 10.1186/s12888-021-03611-6

**Published:** 2021-12-11

**Authors:** Junhan Jiang, Junnan Xu, Li Cai, Li Man, Limin Niu, Juan Hu, Tao Sun, Xinyu Zheng

**Affiliations:** 1 Department of Breast Surgery, First Affiliated Hospital of China Medical University, No. 155 Nanjing North Street, Heping District, Shenyang, 110001 Liaoning China; 2 Department of Breast Medicine, Cancer Hospital of China Medical University, Liaoning Cancer No.44 Xiaoheyan Road, Dadong District, Shenyang, Liaoning 110042 People’s Republic of China; 3 The Fourth Department of Medical Oncology, Harbin Medical University Cancer Hospital, Harbin, 150040 China; 4 Department of Medical Oncology, Anshan Cancer Hospital, Anshan, 114000 China; 5 Breast Cancer Center, The Affiliated Cancer Hospital of Zhengzhou University and Henan Cancer Hospital, Zhengzhou, 450003 China; 6 Department of Breast Cancer Medical Oncology, Hunan Cancer Hospital, Changsha, 410000 China

**Keywords:** Breast cancer, ovarian function suppression, major depressive symptoms, sexual dysfunction, quality of life

## Abstract

**Background:**

Ovarian function suppression (OFS) is indicated in premenopausal women with early or metastasis breast cancer, which may be achieved with similar effect by gonadotropin-releasing hormone agonists (GnRHa) or ovarian ablation (OA). We examined whether there were differences in major depressive symptoms outcomes and its associated factors between gonadotropin-releasing hormone agonists (GnRHa) and ovarian ablation (OA) in premenopausal breast cancer patients.

**Methods:**

Premenopausal breast cancer patients from seven hospitals who received OFS participated in the study between June 2019 and June 2020. The correlated variable was the type of ovarian suppression, categorized as either OA (*n* = 174) or GnRHa (*n* = 389). Major depressive symptoms was evaluated using the Patient Health Questionnaire (PHQ-9), and the Female Sexual Function Index questionnaire was used to assess sexual function.

**Results:**

A total of 563 patients completed the surveys. The mean PHQ-9 sum score was slightly lower in the GnRHa cohort than in the OA cohort (11.4 ± 5.7 vs. 12.8 ± 5.8, *P* = 0.079). There were significantly fewer patients with major depressive symptoms (PHQ-9 ≥ 15) in the GnRHa cohort (31.1% vs. 40.2%, Exp (B)=1.805, P=0.004). Further, breast-conserving surgery and sexual dysfunction were negatively correlated with major depressive symptoms [mastectomy vs. breast-conserving: Exp (B) = 0.461, *P* <0.001;[sexual dysfunction vs. normal: Exp (B) = 0.512, *P* = 0.001].

**Conclusions:**

This is the first study to demonstrate that GnRHa results in more favorable depressive symptoms outcomes than OA. Moreover, most patients preferred alternatives to their OFS treatment. These findings can contribute to improving and alleviating the adverse effects of OFS.

**Supplementary Information:**

The online version contains supplementary material available at 10.1186/s12888-021-03611-6.

## Background

Breast cancer is the most commonly diagnosed cancer and the leading cause of cancer deaths among women worldwide. In China, there has been a marked increase in the breast cancer incidence among younger women [6]. An increasing number of premenopausal Chinese patients aged 45–55 years are being diagnosed with breast cancer, and approximately 6% of patients diagnosed with breast cancer are below 40 years of age [7]. In recent decades, extended adjuvant endocrine therapy, using ovarian function suppression (OFS) has been administered to premenopausal breast cancer patients, with beneficial results for the hormone receptor (HR) -positive population [46]. Depressive symptoms has been identified as one of the most common complications in patients with breast cancer. Depression is correlated with diagnosis, the types of treatment, and adverse effects in breast cancer patients [34]. It has been shown depression might be an critical factor for prognosis and breast cancer recurrence in patients with early breast cancer [44]. Young breast cancer survivors who undergo mastectomy surgery are likely to have worse sexual health, body image and depression compared with women undergoing breast-conserving surgery [36]. However, few studies have compared the impacts of OFS on major depressive symptoms using either ovarian ablation (OA) or gonadotropin releasing-hormone agonists (GnRHa).

OFS, which can eliminate or reduce ovarian estrogen production, was the first endocrine therapy to be investigated for use in premenopausal breast cancer patients. Initially, OFS was achieved through surgical bilateral oophorectomy or ovarian irradiation [24, 27]. However, more recently, GnRHa (also known as luteinizing hormone-releasing hormone agonists) has been used to achieve this outcome [33, 37]. Real-world use of ovarian irradiation for OFS has been limited in China. Consequently, only patients undergoing surgical bilateral oophorectomy or receiving GnRHa were enlisted in this comparative study.

It is now well established that the effect of OFS in reducing the risk of breast cancer relapse or distant metastases is similar to that of the older cyclophosphamide, methotrexate, and fluorouracil regimen [32]. The findings of the Suppression of Ovarian Function Trial (SOFT) indicated that when combined with tamoxifen, OFS had greater benefits for premenopausal women aged <35 years with a high risk of relapse and that a combination of OFS with exemestane increased this benefit [9, 10, 28]. There is an urgent need to explore the use of GnRHa for OFS for protecting ovarian function and improving fertility outcomes following the adverse effects of chemotherapy [3]. Moreover, it is important for clinicians to help patients to understand and protect their ovarian function prior to receiving chemotherapy [1, 38].

A comprehensive analysis of the SOFT results revealed that OFS combined with GnRHa has a similar effect to OA but with clear advantages for premenopausal women [14, 30, 39]. First, GnRHa has reversible effects and can be discontinued if the patient experiences intolerable symptoms. Thus, GnRHa may be preferred over permanent OA by bilateral oophorectomy or ovarian irradiation. All OFS techniques contribute to premature ovarian failure, which has significant consequences, including infertility, sexual dysfunction, and vasomotor symptoms [13, 42]. However, to date, there has been little discussion of emotional disorders, such as major depressive symptoms or sexual dysfunction, associated with the deployment of different OFS strategies. This study examined whether GnRHa is superior to OA in terms of the incidence of major depressive symptoms in premenopausal patients with breast cancer.

## Methods

### Participants

We conducted an anonymous, cross-sectional study between July 2019 and June 2020 at seven hospitals across China. Eligible participants were female breast cancer patients who had either OA by bilateral oophorectomy or GnRHa by one of the following methods: monthly intravenous doses of 3.6 mg of goserelin acetate (Zoladex, AstraZeneca) or monthly intravenous doses of 3.75 mg of leuprorelin acetate (Leuplin, Takeda) or an intramuscular injection of 11.25 mg of leuprorelin acetate once every three months. Indications for ovarian function inhibition in breast cancer patients included premenopausal with either early or metastatic disease. In early breast cancer, endocrine therapy containing OFS is recommended for high-risk premenopausal hormone receptor-positive breast cancer, and should be considered for moderate risk patients. In metastatic hormone receptor-positive breast cancer, all the premenopausal patients should be converted to postmenopausal status and received endocrine therapy. Bilateral ovarian ablation and medical GnRHa had the similar effect and were superior to ovarian radiotherapy. Therefore, either bilateral ovarian ablation or medical GnRHa is indicated in premenopausal women with hormone receptor-positive breast cancer. The eligible participants were invited to complete the Breast Cancer Survivorship Ovarian Function Suppression Survey (OFS-Q5), an online questionnaire that included the Patient Health Questionnaire 9 (PHQ-9), which is an instrument for screening depressive symptoms, and the Female Sexual Function Index (FSFI) questionnaire. The validity and reliability of the Chinese versions of these questionnaires have been previously demonstrated [19, 45, 47]. The study was anonymized to improve the participation rate and accuracy of the responses.

### Included criteria and exclusion criteria

Participants fitting the following criteria were included in our study: (1) a clear diagnosis of breast cancer (tissue or cell diagnosis), (2) age >18 years, and (3) recipient of OFS via surgical bilateral oophorectomy or GnRHa. The exclusion criteria were as follows: (1) refusal to provide informed consent, (2) inability to understand the questionnaires, and (3) undergoing ovarian irradiation. Written informed consent was obtained from each participant, and the study protocols were reviewed and approved by the ethics committee of the Cancer Hospital of China Medical University (protocol RB no. 20190545). The study was conducted in accordance with the principles of the Declaration of Helsinki.

### Questionnaire and data gathering

The questionnaire was divided into four sections. The first section mostly comprised items for obtaining the respondents’ demographic and clinical characteristics, the second section recorded OFS information, and the following two sections respectively evaluated major depressive symptoms and sexual function in both cohorts. Two assistants were trained to develop a knowledge base and communication skills related to OFS and breast cancer. The first assistant explained and confirmed the information, step by step, with the patients, and the second assistant reconfirmed the responses via telephone 3–10 days after the survey’s implementation. The questionnaire was in Chinese, which was the respondents’ native language.

### Depressive symptoms questionnaire

As previously noted ([16]), in PHQ-9 questionnaire, each of the nine items was assigned a score of 0–3, and the scores were then summed to obtain the final score. The following PHQ-9 score ranges have been recommended for determining levels of depression: 0–7: none and/or mild, 8–14: moderate, 15–19: moderate to severe, and 20–27: severe. Lower scores indicate better emotional functioning, whereas a sum score of ≥15 indicates major depression.

### Sexual function questionnaire

Sexual function was quantified using the FSFI questionnaire (Isidori AM et al 2010), which is a 19-item survey instrument that specifically assesses six sexual functioning domains (FSFI-1: desire, FSFI-2: arousal, FSFI-3: lubrication, FSFI-4: orgasm, FSFI-5: satisfaction, and FSFI-6: pain).

### Statistical methods

Data analysis was performed using the IBM SPSS Statistics for Windows, version 23.0 software package (IBM Corp., Armonk, N.Y., USA). Fisher’s exact test was used for categorical data. Differences in age and OFS duration were analyzed using unpaired *t*-tests. The means of the PHQ-9 total scores, FSFI total scores, and FSFI sub-scores were subjected to nonparametric Mann–Whitney *U* tests to determine whether the data were normally distributed. The multivariate analysis was performed with logistics analysis. *P* values < 0.05 indicated statistical significance.

## Results

### Patient characteristics

**Fig. 1:**
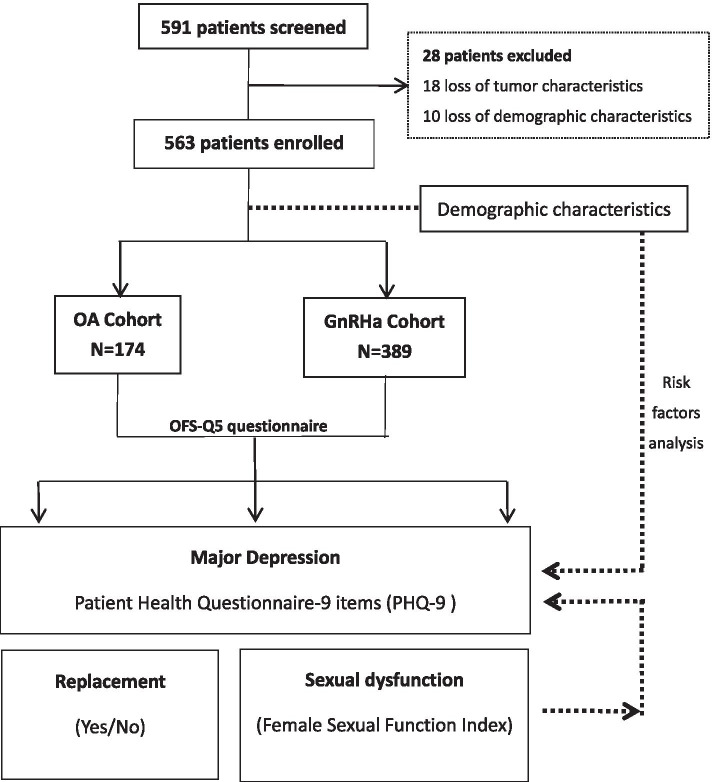
Patients’ profiles in our study. The flow diagram depicts the process of patient enrollment, allocation, and analysis. Abbreviations: OA = ovarian ablation; GnRHa = gonadotropin-releasing hormone agonists; OFS-Q5 = Ovarian Function Suppression Survey 5; PHQ-9 = Patient Health Questionnaire 9

As shown in Fig. 1, the final sample of patients with complete demographic characteristics and PHQ-9/FSFI scores comprised 563 individuals. Of these patients, 174 had undergone OA and 389 were being given GnRHa for OFS. Table 1 lists the main characteristics of the GnRHa and OA cohorts. The patients’ median ages in the OA and GnRHa cohorts were 46.0 and 43.0 years, respectively.

**Table 1:** Baseline social demographics and clinical characteristics in breast cancer patients with ovarian function suppression

Characteristics	Total	Ovarian Ablation OA	GnRH agonist GnRHa	t or χ^2^ value	*P* Value
N	563	174	389		
Age, years					
Median	44	46	43		
Range	22-63	30-57	22-63		
Mean (SD)	42.9 ± 7.7	45.2 ± 6.7	41.8 ± 7.8	5.374	<0.001
Duration of OFS					
Mean (SD), months	25.1 ± 25.4	30.3 ± 29.1	22.8 ± 22.1	3.009	0.003
Educational level				13.570	0.0002
High school or below	268	103 (59.2%)	165 (42.4%)		
College or above	295	71 (40.8%)	224 (57.9%)		
Annual income (RMB)				0.360	0.548
≤ 50,000	398	126 (72.4%)	272 (69.9%)		
> 50,000	165	48 (27.6%)	117 (30.1%)		
Smoking habit				0.597	0.440
Never or little	548	168 (96.6%)	380 (97.7%)		
Mostly	15	6 (3.4%)	9 (2.3%)		
Alcohol drinking habit				1.210	0.271
Never or little	556	170 (97.7%)	386 (99.2%)		
Mostly	7	4 (2.3%)	3 (0.8%)		
Co-morbidity				0.060	0.807
Diabetes/Hypertension	43	14 (8.0%)	29 (7.5%)		
None	520	160 (92.0%)	360 (92.5%)		
Type of Surgery				5.852	0.016
Mastectomy	433	145 (83.3%)	288 (74.0%)		
Breast-conserving	130	29 (16.7%)	101 (26.0%)		
TNM staging				7.505	0.006
I-III	400	110 (63.2%)	290 (74.6%)		
IV	163	64 (36.8%)	99 (25.4%)		
Hormone Receptor Status				12.393	0.0004
ER or PR Positive	526	153 (87.9%)	373 (95.9%)		
ER and PR Negative	37	21 (12.1%)	16 (4.1%)		
HER2 Status				1.256	0.262
Positive	130	35 (20.1%)	95 (24.4%)		
Negative	433	139 (79.9%)	294 (75.6%)		
ET+OFS					
AI	395	115 (66.1%)	280 (72.0%)	0.009	0.924
TAM	86	20 (11.5%)	66 (17.0%)		
Ful	46	19(10.9%)	27(6.9%)		
None	36	20 (11.5%)	16 (4.1%)		

Table 1 shows that the time lapse from the commencement of ovarian suppression was significantly longer in the OA cohort than in the GnRHa cohort (30.3 ± 29.1 months vs. 22.8 ± 22.1 months, *P* = 0.003). The majority of respondents had low annual incomes (77.0%), had estrogen receptor or progesterone receptor positive breast cancers (97.2%), and were human epidermal growth factor receptor-2 (HER2)-negative (76.1%). In addition, 57.9% of the patients in the GnRHa cohort were educated up to or above college level, whereas the corresponding percentage for the OA cohort was only 40.8%. Patients receiving GnRHa favored breast-conserving surgery (26.0% vs. 16.7%, *P* = 0.016).

### Major depressive symptoms in the GnRHa and OA cohorts

The results shown in Table 2 reveal that the mean PHQ-9 sum score of the GnRHa cohort was lower than that of the OA cohort (11.4 ± 5.7 vs. 12.8 ± 5.8, *P* = 0.079). According to the treatment algorithm for depressive symptoms, 36.2% (63/174), 28.2% (49/174), and 12.6% (22/174) of the patients in the OA cohort were respectively categorized in the moderate, major, and severe depressive symptoms groups. Notably, there were significantly fewer patients with major depressive symptoms (PHQ-9 ≥ 15) in the GnRHa cohort than in the OA cohort. A positive correlation was found between major depressive symptoms and OA, and the absolute difference was approximately 9.1 percentage points (31.1% vs. 40.2%, *P* = 0.025). Item-level responses indicated that 15 of the 174 patients who underwent OA and 30 of the 389 patients taking GnRHa experienced suicidal ideation, which is considered as a symptom of major depressive symptoms, but this difference was not statistically significant (8.6% vs. 7.7%, *P* = 0.713).

**Table 2:** Depressive symptoms (PHQ-9) by the type of ovarian function suppression in patients with breast cancer

	Ovarian Ablation (OA) n, (%)	Ovarian function suppression		
		GnRH agonist (GnRHa) n, (%)	t or χ^2^ value	*P* value
Total	174	389		
PHQ-9 scores Median(IQR)	13 (8.0-17.0)	11 (7.0-16.0)		
PHQ-9 scores Range	1-24	0-25		
PHQ-9 scores Mean (SD)	12.8 ± 5.8	11.4 ± 5.7	1.892	0.059
PHQ-9 scores subgroups				
None or mild (0-7), n(%)	40 (23.0)	101 (26.0)		
Moderate (8-14), n(%)	63 (36.2)	167 (42.9)		
Major (15-19), n(%)	49 (28.2)	87 (22.4)		
Severe (20-27), n(%)	22 (12.6)	34 (8.7)		
Major Depression (PHQ≥15), n(%)	71 (40.8)	121 (31.1)	5.033	0.025
Suicidal ideation, n(%)	15 (8.6)	30 (7.7)	0.135	0.713

*PHQ-9* Patient Health Questionnaire - 9 items, *SE *Standard errors, *IQR* Interquartile range (25^th^, 75^th^ percentiles).

### Sexual dysfunction in the GnRHa and OA cohorts

**Fig. 2:**
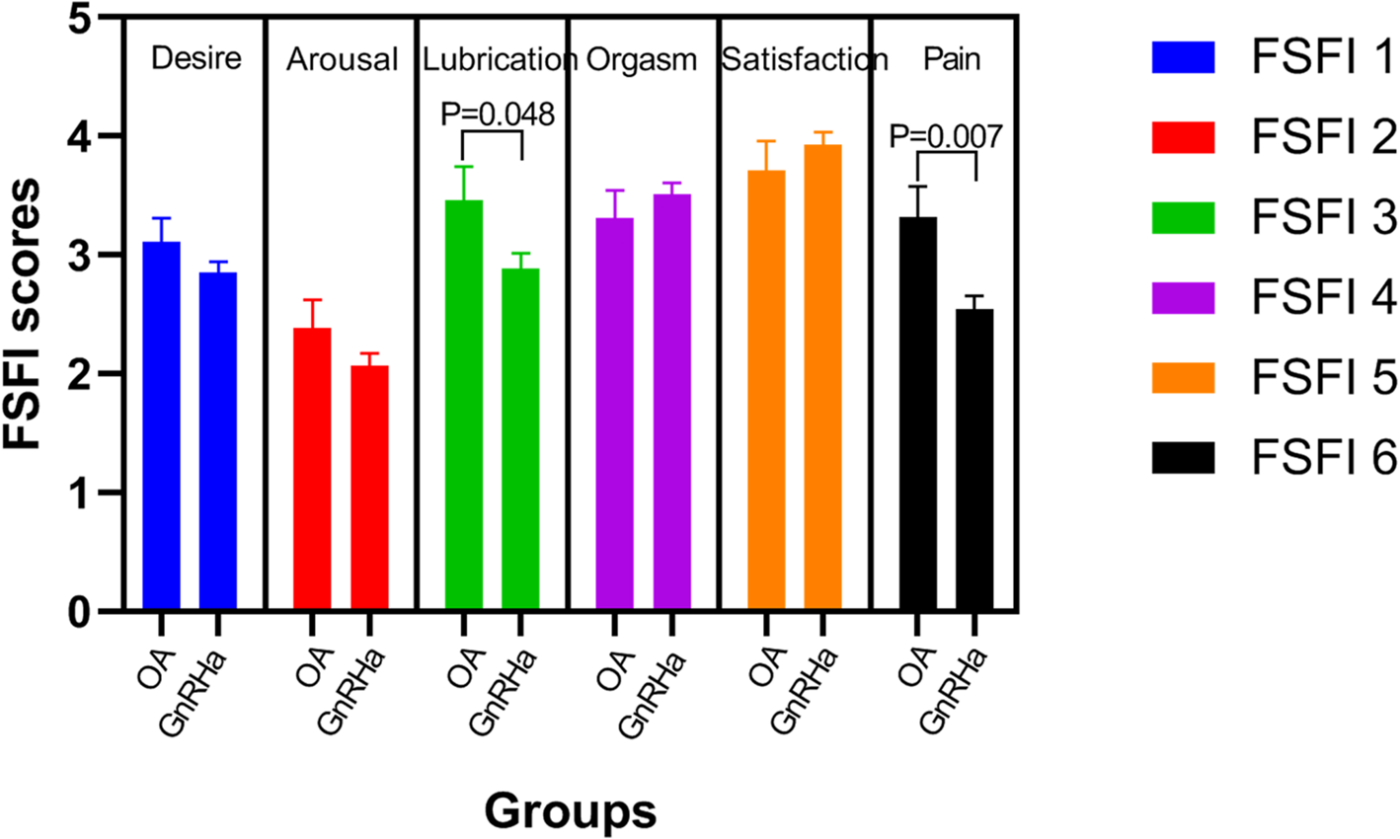
Comparison of responses to sub-items on sexual function provided by women in the GnRHa and OA cohorts. Notes: FSFI-1: desire; FSFI-2: arousal; FSFI-3: lubrication; FSFI-4: orgasm; FSFI-5: satisfaction; FSFI-6: pain. Abbreviations: OA = ovarian ablation; GnRHa = gonadotropin-releasing hormone agonists

As shown in Table 3, patients receiving GnRHa had lower mean and median FSFI scores than patients who had undergone OA (mean: 17.8 ± 8.7 vs. 19.3 ± 8.5, *P* = 0.205; median: 17.8 vs. 19.6). The results for sexual dysfunction, which was defined as FSFI < 23, revealed that there was strong evidence of GnRHa-induced sexual dysfunction; 61.5% of OA patients met the criteria for sexual dysfunction compared with 72.2% of patients receiving GnRHa (*P* = 0.011). According to the data shown in Fig. 2, patients receiving GnRHa had slightly lower scores on most of the FSFI sub-scores compared with the scores of patients who had undergone OA. However, their scores of items in the pain section were significantly lower (FSFI-6 scores: mean ± SD: 3.3 ± 2.2 vs. 2.5 ± 2.2, *P* = 0.007), and there were also significant differences in scores of the item on lubrication (FSFI-3 scores: mean ± SD: 3.5 ± 2.5 vs. 2.9 ± 2.6, *P* = 0.048).

**Table 3:** Sexual function by the type of ovarian function suppression in patients with breast cancer

	Ovarian function suppression			
	Ovarian Ablation (OA) N=174	GnRH agonist (GnRHa) N=389	t or χ^2^ value	*P* value
**FSFI total scores**				
Mean (SD)	19.3 ± 8.5	17.8 ± 8.7		0.205
Median (IQR)	19.6(13.0-26.7)	17.8(12.2-24.3)		
Range	2-34	2-34		
**Sexual dysfunction (FSFI<23), n(%)**	107 (61.5)	281 (72.2)	6.476	0.011

*FSFI* Female sexual function index, *SE *Standard errors, *IQR *Interquartile range (25^th^, 75^th^ percentiles).

### Associated factors for major depressive symptoms in patients with OFS

The data in Table 4 and 5 show that the type of ovarian suppression was closely associated with major depressive symptoms in the univariate and multivariate analyses (OA vs. GnRHa: Exp (B) = 1.805 [95% CI: 1.204–2.705], *P* = 0.004). As noted above, patients receiving GnRHa favored breast-conserving surgery (26.0% vs. 16.7%, *P* = 0.015). Mastectomy breast surgery was negatively correlated with major depressive symptoms in the multivariate analysis (Mastectomy vs breast-conserving: Exp (B)=0.461, 95% CI 0.301-0.707, *P*<0.001). As mentioned in Table 4, patients with fulvestrant combined with OFS had less major depressive symptoms in the univariate analysis. Nonetheless, the combined ET was not significantly correlated with major depressive symptoms (Exp(B)=0.720, 95% CI=0.379-1.366, *P*=0.314) in multivariate analysis in Table 5. Further analysis revealed that sexual dysfunction was negatively associated with major depressive symptoms (sexual dysfunction vs. normal: Exp (B) = 0.512 [95% CI: 0. 348–0.752], *P* = 0.001).

**Table 4:** Clinical characteristics in breast cancer patients with major depressive symptoms

Characteristics	Major depressive symptoms (PHQ≥15)	Normal (PHQ<15)	χ^2^ value	*P* Value
N	191	372		
Age, years			1.624	0.202
≤ 45	118	209		
> 45	73	163		
Educational level			1.519	0.218
High school or below	84	184		
College or above	107	188		
Annual income (RMB)			0.157	0.692
≤ 50,000	133	265		
> 50,000	58	107		
Type of Surgery			13.969	<0.001
Mastectomy	129	304		
Breast-conserving	62	68		
TNM staging			0.019	0.890
I-III	135	265		
IV	56	107		
Hormone Receptor Status			0.039	0.843
ER or PR Positive	179	347		
ER and PR Negative	12	25		
HER2 Status			2.114	0.146
Positive	51	79		
Negative	140	293		
Type of OFS			4.442	0.035
GnRH agonist	121	268		
Ovarian Ablation	70	104		
OFS Time			0.059	0.809
≤ 2 years	116	222		
> 2 years	75	150		
OFS+ET			3.069	<0.080
AI/SERM	128/30	267/56		
Ful	21	25		
Sexual dysfunction			11.146	0.001
No	84	111		
Yes	107	261

**Table 5:** The associated factors on major depressive sympotoms (PHQ-9, scores <15 vs ≥15) by multivariate logistic regression in patients with breast cancer

Characteristics	multivariate				
	B	Wald	P value	Exp(B)	95%CI
Type of Surgery	-0.774	12.570	<0.001		
Breast-conserving				1	
Mastectomy				0.461	0.301-0.707
Type of OFS	-0.590	8.178	0.004		
GnRH agonist				1	
Ovarian Ablation				1.805	1.204-2.705
OFS+ET	-0.329	1.013	0.314		
AI/SERM				1	
Ful				0.720	0.379-1.366
Sexual dysfunction	-0.670	11.620	0.001		
No				1	
Yes				0.512	0.348-0.752

### Alternative to OFS treatment

In the final part of the questionnaire, respondents were asked if they would choose to change to another kind of OFS. For example, “Would you choose OA instead of GnRHa or vice versa?” The majority of the respondents in both cohorts viewed this change positively. Approximately both more than half of the patients in both cohorts opted to change the type of OFS (GnRHa: 62.7% vs. OA: 60.8%). For concerns that costs could affect the final choice, we included the following item: “If cost was not a consideration, would you choose to change to another kind of OFS” In response to this question, the ratio of substitution showed a marked increase to 70.3% for the OA cohort, whereas the increase was not marked for the GnRHa cohort (67.1%). The majority of respondents in both cohorts may have been dissatisfied with their current OFS solution because of depressive symptoms, sexual dysfunction, or an overall decreased quality of life, and the majority of participants were reluctant to receive OA because of its cost.

## Discussion

We conducted a cross-sectional investigation to explore the association between depressive symptoms and the type of OFS (OA or GnRHa) administered to breast cancer patients in seven hospitals. Our results demonstrated that the GnRHa cohort presented with lower levels of major depressive symptoms compared with the OA cohort. This is the first cross-sectional study to provide a direct comparison of levels of serious major depressive symptoms in breast cancer patients undergoing OA and GnRHa. Differing from other studies that used Common Terminology Criteria for Adverse Events (CTCAE) grades to assess the negative effects of OFS, we used PHQ-9 scores in our study. For instance, Moore et al. [25] found that adverse effects identified in early breast cancer patients included eight cases of grade-2 emotional disorders and one case of grade-3 emotional disorders in their OFS cohort compared with three cases of grade-2 emotional disorders in the non-OFS cohort. PHQ-9 questionnaire were used to evaluate major depressive symptoms, as shown in our study, may offer a multidimensional perspective on emotional disorders.

Our results for major depressive symptoms confirmed our hypotheses and endorsed previous findings [2, 19]. Young breast cancer survivors are often at the greatest risk of experiencing major depressive symptoms because of the potential concerns about abrupt menopause and breast disease to relapse, and the incidence of major depressive symptoms among them would be expected considerably higher than the general cohort of the same age range. One meta-analysis of four studies covering 3,373 patients revealed that the difference in depressive symptoms levels between those undergoing OFS and those not undergoing OFS was insignificant (RR: 1.28, 95% CI: 0.94–1.74, *P* = 0.12), with no significant heterogeneity among the studies (*P* = 0.46, *I*^2^ = 0%) [25]. In our investigation, we focused on major depressive symptoms and its potential correlated factors in women with early and metastatic breast cancer. Depression can be linked to both the psychological aspects of social relationships and the physical effects of chemotherapy, can be overwhelming during treatment of metastatic breast cancer [29]. Whether psychiatric disorders will affect the sexual function in breast cancer patients remains an open-ended question with inconsistent results. Young breast cancer survivors who undergo mastectomy surgery have worse sexual health, body image and depression degree compared with women undergoing breast-conserving surgery [36]. In our study, patients with breast-conserving surgery had lower level of major depressive symptoms. The results may indicated major depressive symptoms was correlated with multiple factors and the patients taking OA or mastectomy surgery may be more likely to suffer from major depressive symptoms.

The administration of GnRHa to premenopausal women to reduce estrogen levels can increase the risk of vaginal dryness and dyspareunia [22], which might cause and aggravate sexual dysfunction. Whereas aromatase inhibitors have similar sexual dysfunction effects, they are rarely administered to premenopausal women without OFS. Unlike OA-related sexual dysfunction issues, those associated with GnRHa may be reversible after treatment cessation. However, ovarian failure caused by OA or chemotherapy is permanent [18]. Our results indicated that sexual dysfunction was more common and serious among women in the GnRHa cohort than those in the OA cohort regardless of the duration of the OFS. Notably, the respondents’ mean age within the GnRHa cohort was lower than that of respondents in the OA cohort and more women in the OA cohortmay have reached menopause than those in the GnRHa. Studies on cancer care have reported young survivors have low sexual desire, who are more likely to be distressed by alterations to their appearance than older women [35]. The observed inconsistencies may be attributed to the unequal age distribution within our cohorts [35] and, specifically, spontaneous age-related decline in sexual desire. Previous studies have suggested that the association between tamoxifen and sexual dysfunction remains controversial in premenopausal patients [12, 15, 40, 41]. Fulvestrant may cause lower major depressive symptoms compared with AI or SERM, nonetheless, fulvestrant was only indicated in metastasis breast cancer and the sample was small. We further to pay close attention to sexual dysfunction and major depressive symptoms in patients with diverse combined endocrine therapy.

## Limitations of the study

There were inherent limitations in the cross-sectional design of the study, as indicated below. First, because the survey was anonymized, all treatment information was based on patient recall and could not be re-checked for accuracy using medical records. Therefore, the reporting may not have been sufficiently accurate for making reliable evaluations based on the patients’ responses to the questionnaires alone. A fully prospective evaluation may provide additional information and limit recall bias.

Second, the cross-sectional design constrains us from drawing cause-and-effect conclusions regarding major depressive symptoms and sexual dysfunction. While the reliability and validity of the Chinese versions of the three questionnaires have previously been confirmed [19, 20, 43, 45, 47], we cannot ignore the possibility of social bias given by the methods of delivering the questionnaire and retrieving the responses. Two assistants confirmed the responses with the patients face to face and by telephone in an attempt to reduce the possibility of bias. Additionally, we were unable to evaluate the presence or absence of sufficient ovarian suppression in the GnRHa cohort in our study. Thus, whether or not insufficient ovarian suppression affects major depressive symptoms, sexual dysfunction, and quality of life thus remains unclear. Nevertheless, in spite of its exploratory nature, this study offers some insights into the management of the individual adverse effects of OFS.

Further research will focus on the following three aspects, firstly, whether the different endocrine therapy or chemotherapy will affect the efficacy or depressive symptoms, sexual dysfunction of OFS. Our small sample research indicated the breast cancer patients previous received OFS combined AI suffered from serious vaginal dryness and sexual dysfunction, greatly improved its symptom after changing to OFS combined with tamoxifen. A large and prospective study need to be investigate the correlation and its mechanism. Moreover, the multi-factors investigation may be helpful to provide the optimal OFS type and the patients get the more efficacy benefit and less adverse affects. Last but not least, how to improve major depressive symptoms or sexual dysfunction in patients receiving OFS therapy and presented these symptoms. We have carried out psychological counseling combined with essential oil including jasminum sambac, pelargonium graveolens and leptosermum scoparium in patients with sexual dysfunction. The results will be expected to optimize management of the patients and improve the outcomes.

## Conclusion

We highlight a greater prevalence of major depressive symptoms in patients who had undergone OA than those receiving medically administered GnRHa. This finding indicates that medical GnRHa is a simple, reversible, and preferable therapy for OFS in patients with breast cancer. Further research that examines the closer links between major depressive symptoms and OFS is evidently needed. Moreover, studies should highlight the adverse effects of OFS in premenopausal breast cancer patients. Further, the routine application of a personalized approach seems to be warranted.

## Supplementary Information

**Additional file 1.**

**Additional file 2.**

**Additional file 3.**

## Data Availability

The datasets generated and analysed during the current study are not publicly available due further research but are available from the corresponding author on reasonable request.

## Abbreviations

CTCAE: Common Terminology Criteria for Adverse Events
FSFI: Female Sexual Function Index
GnRHa: gonadotropin-releasing hormone agonists
OA: ovarian ablation
OFS: ovarian function suppression
PHQ-9: Patient Health Questionnaire 9
SOFT: Suppression of Ovarian Function Trialgonadotropin-releasing hormone agonists (GnRHa) and ovarian ablation (OA)
