# Supplementary material for: Major depressive symptoms in breast cancer patients with ovarian function suppression: a cross-sectional study comparing ovarian ablation and gonadotropin-releasing hormone agonists
Source: BMC Psychiatry. 2021 Dec 11;21:624. doi: 10.1186/s12888-021-03611-6 (PMC8666024; doi:10.1186/s12888-021-03611-6)
Supplement: Supplementary file 1 — Additional file 1. [file 12888_2021_3611_MOESM1_ESM.pdf]

# PHQ-9 Scores

### Table Example of PHQ Depression Module for both Diagnostic and Severity Purposes

| **Over the <u>last 2 weeks</u>, how often have you been bothered by any of the following:** | **Not at all** | **Several days** | **More than half the days** | **Nearly every day** |
|---|---|---|---|---|
| | (0) | (1) | (2) | (3) |
| a. Little interest or pleasure in doing things?……………….. | ☐ | ☐ | ☐ | ☐ |
| b. Feeling down, depressed, or hopeless?…………………..… | ☐ | ☐ | ☐ | ☐ |
| c. Trouble falling or staying asleep, or sleeping too much? | ☐ | ☐ | ☐ | ☐ |
| d. Feeling tired or having little energy?........……….....….. | ☐ | ☐ | ☐ | ☐ |
| e. Poor appetite or overeating?...................……………. | ☐ | ☐ | ☐ | ☐ |
| f. Feeling bad about yourself—or that you are a failure or have let yourself or your family down?………………….. | ☐ | ☐ | ☐ | ☐ |
| g. Trouble concentrating on things, such as reading the newspaper or watching television? …………………….. | ☐ | ☐ | ☐ | ☐ |
| h. Moving or speaking so slowly that other people could have noticed? Or the opposite—being so fidgety or restless that you have been moving around a lot more than usual?...........……………………………………….. | ☐ | ☐ | ☐ | ☐ |
| i. Thoughts that you would be better off dead or of hurting yourself in some way? ……………………………………. | ☐ | ☐ | ☐ | ☐ |

**Table PHQ-9 Scores and Proposed Treatment Actions**

| PHQ-9 Score | Depression Severity | Proposed Treatment Actions |
|---|---|---|
| 0 – 4 | None-minimal | None |
| 5 – 9 | Mild | Watchful waiting; repeat PHQ-9 at follow-up |
| 10 – 14 | Moderate | Treatment plan, considering counseling, follow-up and/or pharmacotherapy |
| 15 – 19 | Moderately Severe | Active treatment with pharmacotherapy and/or psychotherapy |
| 20 – 27 | Severe | Immediate initiation of pharmacotherapy and, if severe impairment or poor response to therapy, expedited referral to a mental health specialist for psychotherapy and/or collaborative management |
