# Supplementary material for: Major depressive symptoms in breast cancer patients with ovarian function suppression: a cross-sectional study comparing ovarian ablation and gonadotropin-releasing hormone agonists
Source: BMC Psychiatry. 2021 Dec 11;21:624. doi: 10.1186/s12888-021-03611-6 (PMC8666024; doi:10.1186/s12888-021-03611-6)
Supplement: Supplementary file 2 — Additional file 2. [file 12888_2021_3611_MOESM2_ESM.pdf]

Female Sexual Function Index (FSFI) ©

Subject Identifier ________________________ Date ________________

INSTRUCTIONS: These questions ask about your sexual feelings and responses <u>during the past 4 weeks</u>. Please answer the following questions as honestly and clearly as possible. Your responses will be kept completely confidential. In answering these questions the following definitions apply:

<u>Sexual activity</u> can include caressing, foreplay, masturbation and vaginal intercourse.

<u>Sexual intercourse</u> is defined as penile penetration (entry) of the vagina.

<u>Sexual stimulation</u> includes situations like foreplay with a partner, self-stimulation (masturbation), or sexual fantasy.

CHECK <u>ONLY</u> ONE BOX PER QUESTION.

<u>Sexual desire</u> or <u>interest</u> is a feeling that includes wanting to have a sexual experience, feeling receptive to a partner's sexual initiation, and thinking or fantasizing about having sex.

1. Over the past 4 weeks, how **often** did you feel sexual desire or interest?

   - ☐ Almost always or always
   - ☐ Most times (more than half the time)
   - ☐ Sometimes (about half the time)
   - ☐ A few times (less than half the time)
   - ☐ Almost never or never

2. Over the past 4 weeks, how would you rate your **level** (degree) of sexual desire or interest?

   - ☐ Very high
   - ☐ High
   - ☐ Moderate
   - ☐ Low
   - ☐ Very low or none at all

Sexual arousal is a feeling that includes both physical and mental aspects of sexual excitement. It may include feelings of warmth or tingling in the genitals, lubrication (wetness), or muscle contractions.

3. Over the past 4 weeks, how **often** did you feel sexually aroused ("turned on") during sexual activity or intercourse?

- ☐ No sexual activity
- ☐ Almost always or always
- ☐ Most times (more than half the time)
- ☐ Sometimes (about half the time)
- ☐ A few times (less than half the time)
- ☐ Almost never or never

4. Over the past 4 weeks, how would you rate your **level** of sexual arousal ("turn on") during sexual activity or intercourse?

- ☐ No sexual activity
- ☐ Very high
- ☐ High
- ☐ Moderate
- ☐ Low
- ☐ Very low or none at all

5. Over the past 4 weeks, how **confident** were you about becoming sexually aroused during sexual activity or intercourse?

- ☐ No sexual activity
- ☐ Very high confidence
- ☐ High confidence
- ☐ Moderate confidence
- ☐ Low confidence
- ☐ Very low or no confidence

6. Over the past 4 weeks, how **often** have you been satisfied with your arousal (excitement) during sexual activity or intercourse?

- ☐ No sexual activity
- ☐ Almost always or always
- ☐ Most times (more than half the time)
- ☐ Sometimes (about half the time)
- ☐ A few times (less than half the time)
- ☐ Almost never or never

7. Over the past 4 weeks, how **often** did you become lubricated ("wet") during sexual activity or intercourse?

- ☐ No sexual activity
- ☐ Almost always or always
- ☐ Most times (more than half the time)
- ☐ Sometimes (about half the time)
- ☐ A few times (less than half the time)
- ☐ Almost never or never

8. Over the past 4 weeks, how **difficult** was it to become lubricated ("wet") during sexual activity or intercourse?

- ☐ No sexual activity
- ☐ Extremely difficult or impossible
- ☐ Very difficult
- ☐ Difficult
- ☐ Slightly difficult
- ☐ Not difficult

9. Over the past 4 weeks, how often did you **maintain** your lubrication ("wetness") until completion of sexual activity or intercourse?

- ☐ No sexual activity
- ☐ Almost always or always
- ☐ Most times (more than half the time)
- ☐ Sometimes (about half the time)
- ☐ A few times (less than half the time)
- ☐ Almost never or never

10. Over the past 4 weeks, how **difficult** was it to maintain your lubrication ("wetness") until completion of sexual activity or intercourse?

- ☐ No sexual activity
- ☐ Extremely difficult or impossible
- ☐ Very difficult
- ☐ Difficult
- ☐ Slightly difficult
- ☐ Not difficult

11. Over the past 4 weeks, when you had sexual stimulation or intercourse, how **often** did you reach orgasm (climax)?

- ☐ No sexual activity
- ☐ Almost always or always
- ☐ Most times (more than half the time)
- ☐ Sometimes (about half the time)
- ☐ A few times (less than half the time)
- ☐ Almost never or never

12. Over the past 4 weeks, when you had sexual stimulation or intercourse, how **difficult** was it for you to reach orgasm (climax)?

- ☐ No sexual activity
- ☐ Extremely difficult or impossible
- ☐ Very difficult
- ☐ Difficult
- ☐ Slightly difficult
- ☐ Not difficult

13. Over the past 4 weeks, how **satisfied** were you with your ability to reach orgasm (climax) during sexual activity or intercourse?

- ☐ No sexual activity
- ☐ Very satisfied
- ☐ Moderately satisfied
- ☐ About equally satisfied and dissatisfied
- ☐ Moderately dissatisfied
- ☐ Very dissatisfied

14. Over the past 4 weeks, how **satisfied** have you been with the amount of emotional closeness during sexual activity between you and your partner?

- ☐ No sexual activity
- ☐ Very satisfied
- ☐ Moderately satisfied
- ☐ About equally satisfied and dissatisfied
- ☐ Moderately dissatisfied
- ☐ Very dissatisfied

15. Over the past 4 weeks, how **satisfied** have you been with your sexual relationship with your partner?

- ☐ Very satisfied
- ☐ Moderately satisfied
- ☐ About equally satisfied and dissatisfied
- ☐ Moderately dissatisfied
- ☐ Very dissatisfied

16. Over the past 4 weeks, how **satisfied** have you been with your overall sexual life?

- ☐ Very satisfied
- ☐ Moderately satisfied
- ☐ About equally satisfied and dissatisfied
- ☐ Moderately dissatisfied
- ☐ Very dissatisfied

17. Over the past 4 weeks, how **often** did you experience discomfort or pain during vaginal penetration?

- ☐ Did not attempt intercourse
- ☐ Almost always or always
- ☐ Most times (more than half the time)
- ☐ Sometimes (about half the time)
- ☐ A few times (less than half the time)
- ☐ Almost never or never

18. Over the past 4 weeks, how **often** did you experience discomfort or pain following vaginal penetration?

- ☐ Did not attempt intercourse
- ☐ Almost always or always
- ☐ Most times (more than half the time)
- ☐ Sometimes (about half the time)
- ☐ A few times (less than half the time)
- ☐ Almost never or never

19. Over the past 4 weeks, how would you rate your **level** (degree) of discomfort or pain during or following vaginal penetration?

- ☐ Did not attempt intercourse
- ☐ Very high
- ☐ High
- ☐ Moderate
- ☐ Low
- ☐ Very low or none at all

Thank you for completing this questionnaire

Copyright ©2000 All Rights Reserved
